# Gender differences in respiratory symptoms in 19-year-old adults born preterm

**DOI:** 10.1186/1465-9921-6-117

**Published:** 2005-10-13

**Authors:** Elianne JLE Vrijlandt, Jorrit Gerritsen, H Marike Boezen, Eric J Duiverman, the Dutch POPS-19 Collaborative Study Group*

**Affiliations:** 1 Department of Pediatric Pulmonology, Beatrix Children's Hospital Groningen, UMCG University of Groningen, Hanzeplein 1 9713 GZ Groningen The Netherlands; 2 Department of Epidemiology and bioinformatics, University Medical Center Groningen, University of Groningen, Hanzeplein 1 9713 GZ Groningen The Netherlands

## Abstract

**Objective:**

To study the prevalence of respiratory and atopic symptoms in (young) adults born prematurely, differences between those who did and did not develop Bronchopulmonary Disease (BPD) at neonatal age and differences in respiratory health between males and females.

**Methods:**

*Design*: Prospective cohort study.

*Setting: *Nation wide follow-up study, the Netherlands.

*Participants: *690 adults (19 year old) born with a gestational age below 32 completed weeks and/or with a birth weight less than 1500 g. Controls were Dutch participants of the European Community Respiratory Health Survey (ECRHS).

*Main outcome measures: *Presence of wheeze, shortness of breath, asthma, hay fever and eczema using the ECRHS-questionnaire

**Results:**

The prevalence of doctor-diagnosed asthma was significantly higher in the ex-preterms than in the general population, whereas eczema and hay fever were significant lower. Women reported more symptoms than men. Preterm women vs controls: asthma 13% vs 5% (p < 0.001); hay fever 8% vs 20% (p < 0.001); eczema 10% vs 42% (p < 0.001). Preterm men vs controls: asthma 9% vs 4% (p = 0.007); hay fever 8% vs 17% (p = 0.005); eczema 9% vs 31% (p < 0.001) Preterm women reported more wheeze and shortness of breath during exercise (sob) than controls: wheeze 30% vs 22% (p = 0.009); sob 27% vs 16% (p < 0.001); 19-year-old women with BPD reported a higher prevalence of doctor diagnosed asthma compared to controls (24% vs 5% p < 0.001) and shortness of breath during exercise (43% vs 16% p = 0.008). The prevalence of reported symptoms by men with BPD were comparable with the controls.

**Conclusion:**

Our large follow-up study shows a higher prevalence of asthma, wheeze and shortness of breath in the prematurely born young adults. 19-year-old women reported more respiratory symptoms than men. Compared to the general population atopic diseases as hay fever and eczema were reported less often.

## Background

Neonatal respiratory distress syndrome (RDS), previously called hyaline membrane disease, is mainly seen in preterm infants. The main causative factors leading to respiratory distress in preterm infants are structural immaturity of the lungs, surfactant deficiency and surfactant dysfunction. Most infants recover from RDS. However in infants with a birth weight between 500 and 1500 gram, 3 to 43%, develop chronic lung disease (CLD), also called bronchopulmonary dysplasia (BPD)[1,2].

High rates of respiratory illnesses and other morbidities have been reported in survivors born prematurely in the 1970s and 1980s [3-5]. However reports on long term outcome of respiratory health in adolescents and young adults born prematurely are limited. This can be explained by the continuously changing approach to the treatment of the preterm infant with neonatal RDS, and BPD being a relatively young disorder firstly described about 35 years ago[1]. As the prevalence of both preterm birth and BPD is on the rise and treatment is assessable also in younger infants (from 25 weeks of gestational age), the number of survivors of prematurity will increase[6].

In most reported studies the rates of re-hospitalisation of preterm and/or (extremely) low birth weight infants during the first two years of life, approach or exceed 50%[7]. Overall, respiratory infections are the most common indication for re-hospitalisation[8]. The hygiene- hypothesis states that environmental changes in the industrialised world have lead to reduced microbial contact at an early age and thus resulted in the growing epidemic of atopic diseases as eczema and rhinoconjunctivitis. We were wondering whether the respiratory infections during early life in preterm children resulted in a low prevalence of atopic diseases later.

The aim of the study was to examine the presence or development of respiratory or atopic symptoms in the whole group of (young) adults born prematurely and specifically if premature born did encounter irreversible injuries. In addition, we studied differences in respiratory health at adulthood between those who did and did not develop BPD at neonatal age. Male gender is a risk factor for neonatal RDS, BPD and even death [9-12]. Boys with neonatal RDS seem to have more health problems than girls during the neonatal period [13]. This lead to the question whether we could find differences in respiratory health between young adult males and females.

## Methods

Respiratory health was studied in adults born prematurely in a prospective cohort study. In the early eighties a nation-wide survey was started by the Division of Perinatology of the Dutch Paediatric Association. Information was collected on the incidence of very preterm and very low birth weight infants and subsequently on their outcome on mortality, morbidity and handicap [14,15]. Pre-, peri-, and neonatal data of Dutch infants born alive with a gestational age (GA) below 32 completed weeks and/or with a birth weight less than 1500 g, were collected prospectively. The study ultimately consisted of 1338 infants, constituting 94% of the eligible infants born in 1983 in the Netherlands. All 998 infants surviving the initial hospital stay were enlisted for long term follow-up. Between their birth and the follow up visit in 2002 379 children died, leaving 959 living participants at age 19. BPD was defined as clinical signs of respiratory distress, with an abnormal chest X-ray and an oxygen requirement after 28 days of age.

The European Community Respiratory Health Survey (ECRHS) questionnaire, was mailed to the 959 living participants[16]. This standardised questionnaire was used to assess the prevalence of respiratory symptoms and asthma, in relation to well-known (environmental) risk factors. The Dutch part of the ECRHS data in the youngest age group (20–45 years) was used as control group (644 male and 666 female randomly selected subjects from the general population). The study has been approved by the Ethical Committee of TNO Leiden, the Netherlands.

### Statistical analysis

Data were analysed using the Chi^2^test to compare the prevalence of respiratory symptoms among both ex-preterms and those of the general population. If numbers were too small to use Chi^2^test, we made use of Fisher's exact test. We studied the independent effects of birth weight, gestational age, gender, duration of mechanical ventilation, smoking habits of the parents during the youth of the child and family history of atopic diseases and asthma on the presence of wheeze using multiple logistic regression analysis. Likewise we studied the independent effect of these potential risk factors on the presence of asthma, shortness of breath (with or without exercise), hay fever and eczema respectively. All statistical procedures were performed using SPSS 10.0. P-values less than 0.05 were considered to be significant (2 sided tests).

## Results

The overall response rate was 72% (n = 690). Patient characteristics are shown in Table 1. The non-responders were more likely to be male, have foreign nationalities, lower social-economic status, disabilities and lower school performances at an earlier age than the responders. No differences in birth weight, gestational age or duration of mechanical ventilation were assessed.

**Table 1:** Characteristics participants "Project On Preterm and Small for gestational age infants" (POPS) followed up at the age of 19 year

	total followed-up	Gestational age ≤ 32 weeks	Gestational age >32 weeks
	n = 690	n = 508	n = 182
Gestational age (weeks), mean (range), SD	31 (26–41) ± 2.5	30 (26–32) ± 1.5	34 (32–41) ± 1.5
Birthweight (grams) mean (range), SD	1309 (560–2580) ± 293	1320 (560–2580) ± 325	1277 (600–1495) ± 175
male/female (%)	320/370 (46/54)	244/264 (48/52)	76/106 (42/58)
BPD (yes/no/unknown) (n)	58/505/127	55/362/91	3/143/36
duration mechanical ventilation (days) mean (range), SD	4.5 (0–55) ± 8.1	5.7 (0–55) ± 8.9	1.1 (0–41) ± 3.8

### Premature born

The results of the analyses of the ex-preterms (GA ≤ 32 weeks) are shown in Table 2. The prevalence of doctor-diagnosed asthma and shortness of breath during exercise was significantly higher in the preterm than in the general population, whereas eczema and hay fever were significantly lower. The premature born women reported more symptoms like wheeze than the controls. Women with a birth weight less than 1500 gram (GA >32 weeks) reported more often wheeze and shortness of breath but less allergy and eczema than the female controls. We found no such differences in males.

**Table 2:** Prevalences of symptoms. The children born with a gestational age (GA) ≤ 32 weeks were compared with those born with a GA >32 weeks (birth weight (bw) <1500 g) and with controls according to gender

**Symptom**		**GA ≤ 32 w**	**GA >32 w**	**controls**	**p-value GA ≤ 32 w ** **vs controls**	**p-value GA >32 w ** **vs controls**	**p-value GA ≤ 32 w ** **vs GA >32w **
		**yes (%)**	**yes (%)**	**yes (%)**			
*Have you had wheezing in* * your chest at any time in* * the last twelve months?*	females	79(29.9)	32(30.5)	145(21.8)	**0.009**	**0.05**	0.9
	males	42(17.4)	15(19.7)	122(18.9)	0.6	0.9	0.6
*Have you had this wheezing* * when you did **not **have cold?*	females	35(13.3)	13(12.4)	87(13.1)	0.9	0.8	0.8
	males	25(10.4)	8(10.5)	73(11.3)	0.7	0.8	0.96
*Are you troubled by shortness* * of breath when Hurrying on level ground* * or walking up a slight hill?*	females	60(26.8)	28(34.1)	104(16.3)	**<0.001**	**<0.001**	0.2
	males	19(10.4)	8(12.1)	61(9.9)	0.8	0.5	0.7
*Do you get short of breath* * walking with other people of your* * own age on level ground?*	females	17(7.5)	6(7.5)	11(1.7)	**<0.001**	**0.001**	1.0
	males	5(2.8)	2(3)	9(1.5)	0.23	0.3	1.0
*Do you have to stop* * for breath when walking at your* * own pace on level ground?*	females	12(5.3)	4(5.1)	1(0.2)	**<0.001**	**<0.001**	0.9
	males	6(3.3)	0(0)	4(0.6)	0.004*	0.5	0.3
*Have you ever had asthma?*	females	33(12.7)	7(6.7)	31(4.7)	**<0.001**	0.3	0.1
	males	21(9)	12(16)	28(4.3)	**0.007**	**<0.001**	0.08
*Have you had an attack of asthma* * in the last twelve months?*	females	12(4.6)	4(3.8)	15(2.3)	**0.05**	0.33	0.9
	males	5(2.4)	2(2.7)	2(0.3)	**0.004**	**0.009**	0.7
*Do you have hay fever?*	females	20(7.6)	8(7.5)	135(20.4)	**<0.001**	**0.002**	0.9
	males	23(8.4)	8(10.5)	109(17)	**0.005**	0.15	0.8
*Do you have eczema?*	females	26(9.8)	9(8.5)	276(41.7)	**<0.001**	**<0.001**	0.7
	males	23(9.4)	8(10.5)	196(30.5)	**<0.001**	**<0.001**	0.8

### BPD

111 Children developed BPD (8.2%); 28 of them (25%) died. Boys (n = 72) were more prone to develop BPD than girls (n = 39). The response rate among BPD-patients was 69%. Since the number of BPD patients with a GA >32 weeks was only 3, we decided to analyse the results of the patients with a GA = 32 weeks (table 3). Compared to female controls, 19-year-old females with BPD reported a higher prevalence of doctor-diagnosed asthma, wheeze and shortness of breath during exercise. The BPD males reported significant less hay fever and eczema than the male controls.

**Table 3:** Prevalence of symptoms in participants with a gestational age ≤ 32 weeks with & without BPD and controls according to gender

**Symptom**		BPD	No BPD	controls	p BPD	p no BPD vs	PBPD vs
	
		yes (%)	yes (%)	yes (%)	vs controls	vs controls	no BPD
*Have you had wheezing* * in your chest at any time in* * the last twelve months?*	females	7 (41.1)	58 (29.5)	145 (21.8)	**0.06**	**0.025**	0.3
	males	9 (23.7)	25 (15.3)	122 (18.9)	0.18	0.28	0.2
*Have you had this wheezing* * when you did **not **have cold?*	females	6 (35.3)	23 (11.7)	87 (13.1)	**0.008**	0.6	**0.006**
	males	4 (10.8)	15 (9.2)	73 (11.3)	0.9	0.4	0.7
*Are you troubled by shortness* * of breath when Hurrying on level* * ground or walking up a slight hill?*	females	6 (42.9)	41 (20.8)	104 (16.3)	**0.008**	**0.01**	0.13
	males	3 (12.0)	13 (10.2)	61 (9.9)	0.7	0.9	0.7
*Do you get short of breath* * walking with other people of* * your own age on level ground?*	females	0 (0)	12 (7.1)	11 (1.7)	0.6	**<0.001**	0.6
	males	0 (0)	5 (3.9)	9 (1.5)	0.5	**0.06**	0.6
*Do you have to stop* * for breath when walking at your* * own pace on level ground?*	females	0 (0)	6 (3.6)	1 (0.2)	0.8	**<0.001**	1.0
	males	0 (0)	6 (4.7)	4 (0.6)	0.7	**<0.001**	0.6
*Have you ever had asthma?*	females	4 (23.5)	23 (12)	31 (4.7)	**<0.001**	**<0.001**	0.2
	males	3 (8.1)	13 (8.2)	28 (4.3)	0.28	**0.05**	1.0
*Have you had an attack of* * asthma in the last twelve months?*	females	1 (6.25)	10 (17.2)	15 (2.3)	0.32	**0.007**	1.0
	males	0 (0)	4 (2.5)	2 (0.3)	0.73	**0.004**	0.5
*Do you have hay fever?*	females	1 (5.9)	16 (8.1)	135 (20.4)	0.13	**<0.001**	0.7
	males	1 (2.6)	20 (12)	109 (17)	**0.02**	0.1	0.1
*Do you have eczema?*	females	5 (29.4)	18 (9.1)	276 (41.7)	0.3	**<0.001**	**0.01**
	males	3 (7.9)	13 (7.9)	196 (30.5)	**0.002**	**<0.001**	1.0

### Respiratory symptoms and atopy

In regression analyses dyspnea, asthma, wheeze, dyspnea on exertion, hay fever and eczema were assessed as outcome parameters. Dyspnea was significantly related to long term mechanical ventilation and BPD, maternal asthma and current smoking. An inverse relation was found with gestational age. Asthma was significantly related to maternal asthma. Wheeze was significantly related to female gender and current smoking habits and tended to be related to maternal smoking during the youth of the participant. Shortness of breath during exercise was related to female gender and smoking in the past. We found no significant associations of birth weight, gestational age, duration of mechanical ventilation, gender, smoking habits or BPD to hay fever and eczema (see table 4). Young adults with recurrent respiratory infections in infancy reported more asthmatic symptoms than those without respiratory infections (p < 0.001). No significant differences were found between recurrent respiratory infections and hayfever or eczema. Young adults with sepsis during the neonatal period reported less hayfever than those without sepsis (p = 0.03), but no significant differences were found between sepsis and asthma or eczema.

**Table 4:** Odds ratios (95% confidence intervals) for respiratory symptoms, hay fever and eczema, determined by multiple regression analysis. Significant relations are printed in bold. Birth weight, gestational age, duration of mechanical ventilation and smoking habits are entered as categorical covariates.

		**dyspnea**	**asthma**	**wheeze**	SOBDE*	**hayfever**	**eczema**
**birth weight (gram)**	**500–1000**	0.4 (0.2–1.1)	0.6 (0.2–2.4)	1.6 (0.7–4.0)	0.6 (0.2–1.9)	1.2 (0.3–4.4)	1.2 (0.3–4.5)
	**1000–1500**	0.5 (0.3–1.2)	0.8 (0.3–2.0)	1.7 (0.8–3.6)	1.0 (0.4–2.3)	1.4 (0.5–3.6)	1.5 (0.6–4.3)
**Gestational age (weeks)**	**till 28**	**0.4 (0.2–0.9) †**	0.8 (0.2–2.6)	0.9 (0.4–1.9)	1.1 (0.5–2.7)	1.3 (0.4–4.2)	0.9 (0.3–2.7)
	**28–31**	**0.5 (0.2–0.9) †**	1.1 (0.4–2.6)	1.0 (0.6–1.9)	0.7 (0.3–1.4)	1.2 (0.5–2.9)	0.9 (0.4–2.3)
**Mechanical ventilation (days)**	**1–7 days**	1.1 (0.6–216)	1.3 (0.6–3.1)	1.0 (0.5–1.9)	1.1 (0.5–2.2)	1.4 (0.6–3.2)	1.21 (0.5–2.8)
	**8–28 days**	0.9 (0.4–2.2)	0.3 (0.1–1.5)	1.2 (0.6–2.6)	0.7 (0.3–1.9)	0.7 (0.2–2.3)	1.1 (0.4–3.4)
	**>28**	**5.2 (1.2–23.3) †**	0.3 (0.0–4.0)	1.6 (0.4–6.9)	0.2 (0.2–2.7)	0.7 (0.1–7.7)	0.4 (0.0–3.7)
**female gender**		1.6 (1.0–2.7)	1.2 (0.6–2.3)	**2.0 (1.2–3.2) †**	**3.8 (2.0–7.3) ‡**	0.7 (0.1–7.7)	1.3 (0.7–2.5)
**Maternal smoking**		1.1 (0.6–1.9)	1.6 (0.7–3.5)	1.6 (1.0–2.8)	1.3 (0.7–2.5)	0.6 (0.3–1.3)	1.0 (0.5–2.1)
**Maternal asthma**		**2.5 (1.2–5.4) †**	**4.2 (1.8–10.5) ‡**	1.3 (0.6–2.9)	1.6 (0.6–4.1)	1.8 (0.6–5.2)	2.0 (0.7–5.3)
**BPD**		**3.1 (1.2–8.2) †**	3.1 (0.7–14.6)	1.5 (0.6–3.6)	2.0 (0.6–6.9)	0.4 (0.1–2.3)	2.4 (0.8–7.4)
**Smoking participant**	**past**	1.2 (0.6–2.5)	0.6 (0.2–1.7)	0.9 (0.4–1.9)	**2.2 (1.0–4.8) †**	1.5 (0.6–3.6)	1.4 (0.6–3.4)
	**"party"**	0.5 (0.1–1.5)	0.9 (0.3–2.9)	1.9 (0.8–4.0)	0.9 (0.3–2.5)	0.3 (0.0–2.3)	1.3 (0.5–3.8)
	**daily**	**2.7 (1.5–5.1) ‡**	0.4 (0.1–1.1)	2.6 (1.5–4.6) ‡	1.5 (0.7–2.9)	1.6 (0.7–3.7)	1.0 (0.4–2.5)

*SOBDE = shortness of breath during exercise, † p < 0.05, ‡ p = 0.001

## Discussion

In this long-term follow-up of ex-preterms into adulthood we found a higher prevalence of asthma, wheezing and shortness of breath during exercise in the ex-preterms (especially the women) compared to the general population. Atopy (i.e. hay fever, rhino-conjunctivitis and atopic dermatitis) was significantly lower in the ex-preterms compared with the controls. In this study, we did not perform lung function, skin prick or RAST tests to confirm the diagnoses. However, the relation between subject reported symptoms on the basis of the used ECHRS questionnaire and lung function is studied earlier. Subject reported symptoms were related to impaired lung function and to increased variability of peak flow[17].

Long-term reports on respiratory health in infants born prematurely are limited and contradictory. Respiratory health of preterm children of birth weight ≤ 1500 g at 14 years of age has been reported to be comparable to that of term controls [18]. Others found that infants born prematurely with and without a history of neonatal RDS, but who did not develop BPD, have an increased prevalence of airway hyperreactivity compared to full term controls which can persist into early adult life [3,4]. At school age bronchial obstruction and increased bronchial responsiveness have been demonstrated in prematurely born children [19].

### Preterm birth and asthma

The pathophysiology of neonatal RDS is not completely understood, but it has been demonstrated that factors such as mechanical ventilation and oxygen lead to an inflammatory process, which could result in an early Th1- response. Moreover, in most reported studies the rates of re-hospitalisation of preterm and/or (extremely) low birth weight infants during the first two years of life, approach or exceed 50%[7]. Respiratory illnesses and especially respiratory infections are the most common indication for re-hospitalisation in this patient group. Also in our cohort the re-hospitalisation-rate in early childhood was high (34%)[14]. In contrast re-admission rates for normal birth weight infants are much lower (about 20%)[7]. Asthma is often characterised by symptoms like shortness of breath and wheeze; reversible airway obstruction; airway hyper-responsiveness and airway inflammation. In children and young adults, asthma is associated with atopy through IgE-dependent mechanisms, and airway-inflammation is partly related to helper T type 2 (Th2) lymphocytes and eosinophil mediation [20]. Preterm born adults report asthma-like symptoms, but less allergy compared to controls. Decreased risk of atopy is also found in a Finnish prospective birth cohort study comparing term and preterm adults: high gestational age increased the risk of atopy at the age of 31[21]. The early Th1- response, in combination with serious infections in the first two years of life, could be an explanation for the lower prevalence of atopy, which is in line with the hygiene-hypothesis [22]. Others found that children who were septic in the neonatal period were less likely to have asthma[23]. We could not confirm this. However, young adults who were septic during the neonatal period did report less hayfever. In our study, adults with recurrent respiratory infections in infancy did not report *less *but *more *asthma. This is remarkable considering that early exposure to endotoxins or other allergens enhance Th1-type cytokine responses tip the balance away from Th2-type responses that favour the development of allergic diseases including asthma[24]. The high rate of respiratory symptoms might be due to sustained increased vulnerability of the immature airways in a way that mimics asthma, but is not exactly the same.

### Gender

Male gender is a risk factor for neonatal RDS and BPD [9-11]. Boys with neonatal RDS seem to have more health problems than girls during the neonatal period and school age[13,23]. However, *long-term *outcome shows gender differences in e.g. school-performances, but not in respiratory health. We found that particularly women reported symptoms as wheeze and shortness of breath. In the 'general' population both incidence and prevalence of wheeze and asthma is higher in males than in females until the age of 16 year [25,26]. In adulthood, asthma occurs more frequently among women[25,26]. The observed variation between males and females in the general population has partly been explained by dys-synnaptic lung growth: the independent growth of the airways in comparison with the lung parenchyma and air spaces. In girls, growth of the airways is proportional to growth of lung parenchyma, whereas in boys growth of the airways lags behind that of lung parenchyma, causing a discrepancy between airway and lung size [27]. Different pubertal patterns of thoracic growth between the sexes results in an approximately 25% higher lung function in males than in females of identical height at the end of puberty. We speculate that a similar process takes place in the preterm born population, although the underlying mechanism is not understood. Another explanation might be that large individual differences exist in physical symptom reports. Women may require a greater amount of cognitive analysis (and thus more attention) to make judgements about physical symptoms compared to men[28].

There have been few reports of respiratory health during exercise. Our finding of a high percentage of participants that reported shortness of breath during exercise, is in agreement with a study showing low oxygen consumption in low birth weight children compared to children with a normal birth weight [29]. The authors suggested that extremely low birth weight children have a lower level of fitness than controls.

### BPD

Airway obstruction and airway hyper-reactivity persisted in children and adolescents with BPD [3,4,30]. Long-term studies in children who had BPD as infants showed persisting lung function abnormalities consisting of airway obstruction, airway hyper-reactivity, and hyperinflation[5,31]. Both BPD and asthma are characterised by increased smooth muscle contraction and symptoms of both diseases are therefore perhaps difficult to distinguish. As stated above, airway inflammation is an important feature in children and adults with asthma. Studies showed that inflammation plays an important role in the pathogenesis of BPD. Contrary to asthma, however, the BAL-fluid reflects a Th1-cell subtype[32,33]. Even more than preterm infants without BPD, infants with BPD are likely to be re-hospitalised early in childhood with a respiratory illness[8,34]. The same mechanism as described above could be an explanation for the low prevalence of hay fever and eczema, despite the asthma-like symptoms.

### Analysis of risk factors for respiratory symptoms

The regression analysis confirmed the association between dyspnea and respectively long-term mechanical ventilation, BPD and smoking of the participant. We expected to find high risks for respiratory symptoms in the young adults with a (very) low birth weight or born (very) prematurely due to the immaturity of the airways at birth. However, the degree of prematurity or dysmaturity did not increase the risk at all. As a matter of fact, the risk for dyspnea was even lower in the children born very prematurely. In seeking to understand this we speculate that these young adults have a bias toward symptom detection and the feeling of distress because they are used to physical limitations. Future research should investigate the extend to which physical symptoms correlate with lung function abnormalities.

A limitation of our study might be that the age range of the preterms and the general population sample is not exactly the same. However, the prevalence of respiratory symptoms is probably increasing with age. Therefore, the differences might even be more obvious when the results of young adults born prematurely could be compared with peers from the general population. As the complete cohort was inhomogeneous in the sense that it consisted of either preterm or small for gestational age infants, we choose to analyse the data of the preterm children (GA ≤ 32 weeks). The possibility that symptoms will disappear and that ex-preterms will "grow out" of their disease *after *adolescence is likely to be very small because the lungs stop growing and developing after that age. It might even be possible that symptoms come back or become more severe during adulthood, as has been observed in long-term follow-up of asthma[35].

## Conclusion

Our study clearly demonstrated that more than a third of young adults born preterm suffer from respiratory symptoms (higher prevalence of asthma, wheeze and shortness of breath) and need more medical care than peers. Not only paediatricians, but also family doctors and chest physicians should be aware of this 'new' group of patients in which respiratory symptoms will never disappear. Especially women seem to be more vulnerable on their way to adulthood and report more respiratory symptoms than controls. Future research should investigate to what extend physical symptoms correlate with lung function abnormalities. On the other hand, our findings are encouraging because a lot of young adults born preterm, survive with no or only minor respiratory problems and compared to the general population atopic diseases as hay fever and eczema were reported less often.

## Competing interests

The author(s) declare that they have no competing interests.

## Authors' contributions

**EV **participated in design and co-ordination of the study, analysis & interpretation of the data and drafting of the article.

**JG **and **ED **have made substantial contributions to the design of the study, the interpretation of data and drafting the article

**HB **has made substantial contributions to (statistical) analysis, interpretation of the data and drafting the article

All authors read and approved the final manuscript

## Funding

major funding was provided by the "Stichting Astmabestrijding"

## Note

* Participants of the Dutch POPS-19 Collaborative Study Group:

TNO Prevention and Health, Leiden (ETM Hille, CH de Groot, H Kloosterboer-Boerrigter, AL den Ouden, A Rijpstra, SP Verloove-Vanhorick, JA Vogelaar); Emma Children's Hospital AMC, Amsterdam (JH Kok, A Ilsen, M van der Lans, WJC Boelen-van der Loo, T Lundqvist, HSA Heymans); University Hospital Groningen, Beatrix Children's Hospital, Groningen (EJ Duiverman, WB Geven, ML Duiverman, LI Geven, EJLE Vrijlandt); University Hospital Maastricht, Maastricht (ALM Mulder, A Gerver); University Medical Center St Radboud, Nijmegen (LAA Kollée, L Reijmers, R Sonnemans); Leiden University Medical Center, Leiden (JM Wit, FW Dekker, MJJ Finken); Erasmus MC – Sophia Children's Hospital, University Medical Center Rotterdam (N Weisglas-Kuperus, MG Keijzer-Veen, AJ van der Heijden, JB van Goudoever); VU University Medical Center, Amsterdam (MM van Weissenbruch, A Cranendonk, HA Delemarre-van de Waal, L de Groot, JF Samsom); Wilhelmina Children's Hospital, UMC, Utrecht (LS de Vries, KJ Rademaker, E Moerman, M Voogsgeerd); Máxima Medical Center, Veldhoven (MJK de Kleine, P Andriessen, CCM Dielissen-van Helvoirt, I Mohamed); Isala Clinics, Zwolle (HLM van Straaten, W Baerts, GW Veneklaas Slots-Kloosterboer, EMJ Tuller-Pikkemaat); Royal Effatha Guyot Group, Zoetermeer (MH Ens-Dokkum); Association for Parents of Premature Babies (GJ van Steenbrugge).
